# Endogenous and exogenous regulation of gamma-secretase

**DOI:** 10.1186/1750-1326-8-S1-O10

**Published:** 2013-09-13

**Authors:** Yueming Li

**Affiliations:** 1 Molecular Pharmacology and Chemistry Program, Memorial Sloan-Kettering Cancer Center, New York, USA

## 

The gamma-secretase complex is an intramembrane aspartyl protease comprised of at least four known subunits: Presenilin (PS), Nicastrin (Nct), Aph, and Pen2. Presenilin is the catalytic subunit of the complex. Mutations in PS1 and PS2, which lead to familial early-onset Alzheimer disease, alter the activity and specificity of gamma-secretase. Investigation of gamma-secretase specificity and development of gamma-secretase based therapies have been a huge challenge because of its nature of intramembranal catalysis and macromolecular complex as well as cleavage of multiple substrates. We have developed an integrated approach of biochemistry, chemical biology and cell biology for the study of gamma-secretase structure and function. We will discuss how to apply this approach to elucidate the role endogenous and exogenous factors in regulation of gamma-secretase. We will focus on the development of chemical probes and utilize them to define the mechanism of action of gamma-secretase modulators.

